# Peri-Extubation Dexamethasone to Prevent Post-extubation Airway Obstruction in Critically Ill Children: A Systematic Review of Randomized Trials Across Heterogeneous Populations, Regimens, and Outcomes

**DOI:** 10.7759/cureus.115518

**Published:** 2026-08-31

**Authors:** Abdulelah F Alshehri, Dana Naif Al-Dahmashi, Watin Jazzaa Alanazi, Reem Taif Al-Dahmashi, Abir Shiban Alenezi, Wasan Saud Alanazi, Farah Safaq Al-Dahmashi, Joud Falah Alanazi, Joory Ateah Alruwili, Sarah Nasser Almuhaysin, Shahad Abdullah Alruwaili, Yasmin Mibrad ALenezi, Shelian Alnomsi, Mohammed Ghazai Aloufi, Abdulrahman A Almazrou

**Affiliations:** 1 Clinical Research, Independent Research, Riyadh, SAU; 2 College of Medicine, Northern Border University, Arar, SAU; 3 Department of Pulmonary Medicine, King Faisal Specialist Hospital & Research Centre, Riyadh, SAU; 4 College of Medicine, Imam Mohammad Ibn Saud Islamic University, Riyadh, SAU; 5 Department of Ophthalmology, Imam Mohammad Ibn Saud Islamic University, Riyadh, SAU

**Keywords:** dexamethasone, extubation failure, mechanical ventilation, pediatric intensive care, post-extubation stridor

## Abstract

Peri-extubation dexamethasone is used to prevent post-extubation upper airway obstruction in critically ill children, but pediatric randomized trials differ substantially in population selection, dosing, timing, and outcome definitions. We systematically reviewed randomized controlled trials evaluating peri-extubation dexamethasone in mechanically ventilated children. PubMed/MEDLINE, Scopus, and Web of Science were searched from inception to June 22, 2026. Nine trials randomized 1,063 children, with 949 included in reported analyses. Because comparator architecture, dexamethasone exposure, extubation context, outcome constructs, and follow-up windows were not clinically comparable, findings were synthesized narratively rather than pooled. Among placebo-controlled trials with interpretable pairwise binary airway outcomes, no trial provided statistically significant evidence of a reduction in the principal airway obstruction outcome. A 24-hour versus 6-hour regimen comparison reported less clinically significant airway obstruction with the 24-hour regimen, but the groups also differed in pre-extubation dose count and cumulative exposure, preventing attribution to timing alone. A low-dose versus high-dose trial did not establish non-inferiority of the lower-dose regimen. Reintubation outcomes showed no consistent treatment effect and were limited by imprecision and variable attribution. Adverse event ascertainment was limited. Seven trials raised some concerns, and two were at high overall risk of bias. Current randomized evidence does not define a single optimal peri-extubation dexamethasone regimen. Standardized eligibility criteria, explicit reporting of actual pre-extubation exposure, consistent airway outcome definitions, and prospective attribution of reintubation are needed before an optimal prophylactic strategy can be established.

## Introduction and background

Post-extubation upper airway obstruction is a recognized complication of invasive mechanical ventilation in critically ill children and has been documented prospectively after prolonged intubation in the pediatric intensive care unit [1]. Airway injury is among the complications attributable to mechanical ventilation in this population [2]. The clinical entity is broader than audible stridor alone. Children may develop increased work of breathing, chest wall retractions, or impaired oxygenation without a prominent inspiratory sound, making post-extubation stridor a clinical manifestation of post-extubation upper airway obstruction rather than a synonymous term.

Young children are particularly vulnerable because relatively small reductions in airway caliber can substantially increase airflow resistance and the work of breathing. The subglottic airway is susceptible to circumferential mucosal injury and edema related to endotracheal intubation. When obstruction becomes clinically important, management may escalate from observation to nebulized epinephrine, non-invasive respiratory support, and reintubation. Extubation failure after planned extubation is associated with prolonged intensive care stay and additional morbidity, and upper airway obstruction is among its recognized causes [3]. Extubation failure may also result from mechanisms unrelated to upper airway edema. Respiratory muscle weakness, for example, is independently associated with pediatric extubation failure and would not be expected to respond to corticosteroid prophylaxis [4].

Because post-intubation laryngeal edema has an inflammatory component, corticosteroid administration before extubation has a plausible mechanistic rationale. Dexamethasone is commonly used because of its potent anti-inflammatory activity, minimal mineralocorticoid effect, and relatively prolonged duration of action. A Cochrane review evaluating corticosteroid prophylaxis across neonates, children, and adults did not establish a definitive approach for pediatric practice [5]. Evidence in adults is more consistent, with meta-analysis of randomized trials showing reductions in post-extubation stridor and reintubation, particularly when corticosteroids are administered several hours before extubation in patients considered at increased risk [6].

Pediatric evidence has been more difficult to translate into a single treatment strategy. A systematic review and meta-analysis of pediatric trials reported reductions in post-extubation upper airway obstruction and extubation failure while also identifying heterogeneity across populations and corticosteroid regimens [7]. A subsequent network meta-analysis grouped dexamethasone regimens according to dose and timing and suggested that earlier initiation may be more important than dose in preventing upper airway obstruction, although the certainty of the evidence was low and estimates of reintubation remained very uncertain [8]. 

Previous syntheses have primarily focused on pooled efficacy or selected treatment modifiers such as dose and timing. Less attention has been given to the broader sources of non-comparability across randomized trials, including population eligibility, cumulative and pre-extubation dexamethasone exposure, endpoint construction, outcome assessment windows, and rescue treatment practices. Characterizing these differences is important for determining how directly individual randomized comparisons can inform clinical practice. We therefore conducted a systematic review of randomized trials of peri-extubation dexamethasone for the prevention of post-extubation airway obstruction in critically ill children, examining heterogeneity across trial populations, dexamethasone regimens, and clinically relevant airway outcomes.

## Review

Methods

Study Design and Reporting Standards

This systematic review evaluated randomized controlled trials (RCTs) assessing peri-extubation dexamethasone for the prevention of post-extubation airway obstruction in critically ill children. The review was conducted and reported in accordance with the Preferred Reporting Items for Systematic Reviews and Meta-Analyses (PRISMA) 2020 statement [9]. The review was not prospectively registered.

Literature Search Strategy

PubMed/MEDLINE, Scopus, and Web of Science were searched from database inception through June 22, 2026. The search combined subject headings, where applicable, and free-text terms related to pediatric populations, extubation, post-extubation airway obstruction or stridor, dexamethasone or corticosteroids, and RCTs. Search syntax was adapted to the indexing structure and interface of each database. Reference lists of eligible studies were manually screened to identify additional potentially relevant reports. Duplicate records were removed before screening. The core search framework is presented in Table 1.

**Table 1:** Search Framework Used for the Systematic Review

Search component	Search terms
Population/condition	(child* OR pediatric* OR paediatric* OR infant* OR PICU) AND ("post-extubation" OR "postextubation" OR "peri-extubation" OR "extubation failure") AND (stridor OR "airway obstruction" OR "laryngeal edema" OR "upper airway obstruction")
Intervention	dexamethasone OR corticosteroid* OR steroid*
Study design	random* OR "randomized controlled trial" OR "randomised controlled trial"
Combined search framework	[(child* OR pediatric* OR paediatric* OR infant* OR PICU) AND ("post-extubation" OR "postextubation" OR "peri-extubation" OR "extubation failure") AND (stridor OR "airway obstruction" OR "laryngeal edema" OR "upper airway obstruction")] AND (dexamethasone OR corticosteroid* OR steroid*) AND (random* OR "randomized controlled trial" OR "randomised controlled trial")

Eligibility Criteria

Eligibility criteria were defined according to the Population, Intervention, Comparison, Outcome, and Study design (PICOS) framework before study selection. Eligible studies enrolled critically ill children receiving invasive mechanical ventilation and undergoing planned or repeat extubation. Trials enrolling both adults and children were eligible only when pediatric data were separately extractable.

Eligible interventions consisted of peri-extubation dexamethasone initiated before extubation, irrespective of dose, timing, number of doses, or cumulative regimen. Comparators included placebo, no prophylaxis or standard care, or an alternative dexamethasone regimen. Only RCTs reporting an eligible post-extubation airway outcome were included.

The primary review outcome was post-extubation upper airway obstruction or stridor according to the definition used by each trial. Secondary outcomes included airway obstruction severity scores, rescue treatment requirements, reintubation or extubation failure, and adverse events. Detailed eligibility criteria are presented in Table 2.

**Table 2:** Inclusion and Exclusion Criteria for Study Eligibility

Eligibility criterion	Inclusion criteria	Exclusion criteria
Population	Critically ill children requiring invasive mechanical ventilation and undergoing planned or repeat extubation; mixed adult and pediatric trials when pediatric data were reported separately	Adult-only populations or mixed populations without separately extractable pediatric data
Extubation context	First or repeat extubation attempts evaluating peri-extubation dexamethasone prophylaxis	Extubation contexts not addressing the predefined review question
Intervention	Peri-extubation dexamethasone initiated before extubation, at any dose, timing, number of doses, or cumulative regimen	Interventions not including eligible peri-extubation dexamethasone
Comparator	Placebo, no prophylaxis or standard care, or an alternative dexamethasone regimen	Comparators outside the predefined review question
Outcomes	Post-extubation airway obstruction or stridor, laryngeal edema, severity scores, rescue treatment requirement, reintubation or extubation failure, or adverse events	No extractable eligible post-extubation airway outcome
Study design	Randomized controlled trials	Non-randomized studies, observational studies, case series, reviews, or protocols without eligible outcome data
Dataset	Unique eligible randomized datasets	Duplicate or overlapping reports without additional eligible data

Study Selection and Data Extraction

Titles and abstracts were screened for potential eligibility, followed by full-text assessment of potentially relevant reports against the predefined eligibility criteria. Reasons for exclusion at the full-text stage were recorded.

Data were extracted using a standardized extraction framework. Extracted variables included author and publication year, country and clinical setting, trial design and blinding, randomized and analyzed sample sizes, participant age and relevant population characteristics, intubation and extubation characteristics, dexamethasone dose, timing, number of doses and cumulative exposure, comparator characteristics, outcome definitions and assessment instruments, outcome assessment windows, rescue treatment practices, reintubation or extubation failure outcomes, and adverse event data. When multiple reports referred to the same randomized dataset, information was considered together to avoid duplicate inclusion while retaining relevant supplementary or corrected data.

Risk-of-Bias Assessment

Risk of bias was assessed using the Cochrane Risk of Bias 2 (RoB 2) tool [10]. The assessed domains were bias arising from the randomization process, bias due to deviations from intended interventions, bias due to missing outcome data, bias in measurement of the outcome, and bias in selection of the reported result. Domain-level and overall judgments were classified as low risk, some concerns, or high risk of bias.

Because RoB 2 assessments are result-specific, judgments were based on the principal post-extubation airway obstruction result used in the review for each trial rather than on the study. Risk-of-bias judgments were summarized using a traffic light figure.

Data Synthesis and Statistical Analysis

Clinical and methodological comparability was assessed before considering quantitative synthesis. Meta-analysis was not performed because the available randomized comparisons differed in comparator architecture, population eligibility, dexamethasone dose and regimen, extubation context, outcome definitions and thresholds, assessment instruments, and follow-up windows, such that a common pooled effect estimate was not considered clinically interpretable.

Findings were therefore synthesized narratively and organized according to comparator architecture: placebo or no prophylaxis comparisons, head-to-head dexamethasone regimen comparisons, and repeat extubation evidence. Differences in population selection, dexamethasone exposure, endpoint construction, rescue-treatment practices, and outcome ascertainment were additionally examined as sources of non-comparability across trials.

Study-level effect estimates, including risk ratios, odds ratios, absolute risk differences, and numbers needed to treat, were extracted as reported when available. Estimates obtained from published corrections were identified as such. When descriptive effect measures were derived directly from reported randomized arm-level data, they were explicitly identified and were not pooled. For trials reporting different analysis populations, including intention-to-treat and per-protocol analyses, the relevant population and post-randomization exclusions were explicitly described.

No formal statistical heterogeneity testing, meta-regression, publication-bias testing, or funnel-plot analysis was performed because no quantitative synthesis was undertaken and the number of eligible trials was small.

Results

Study Selection

The database search identified 237 records. After removal of 72 duplicates, 165 records underwent title and abstract screening, of which 134 were excluded. Thirty-one full-text reports were retrieved and assessed for eligibility, and 22 were excluded, most commonly because of non-randomized study designs, ineligible populations, or interventions outside the peri-extubation dexamethasone scope. Nine randomized trials were included in the systematic review (Figure 1).

**Figure 1:**
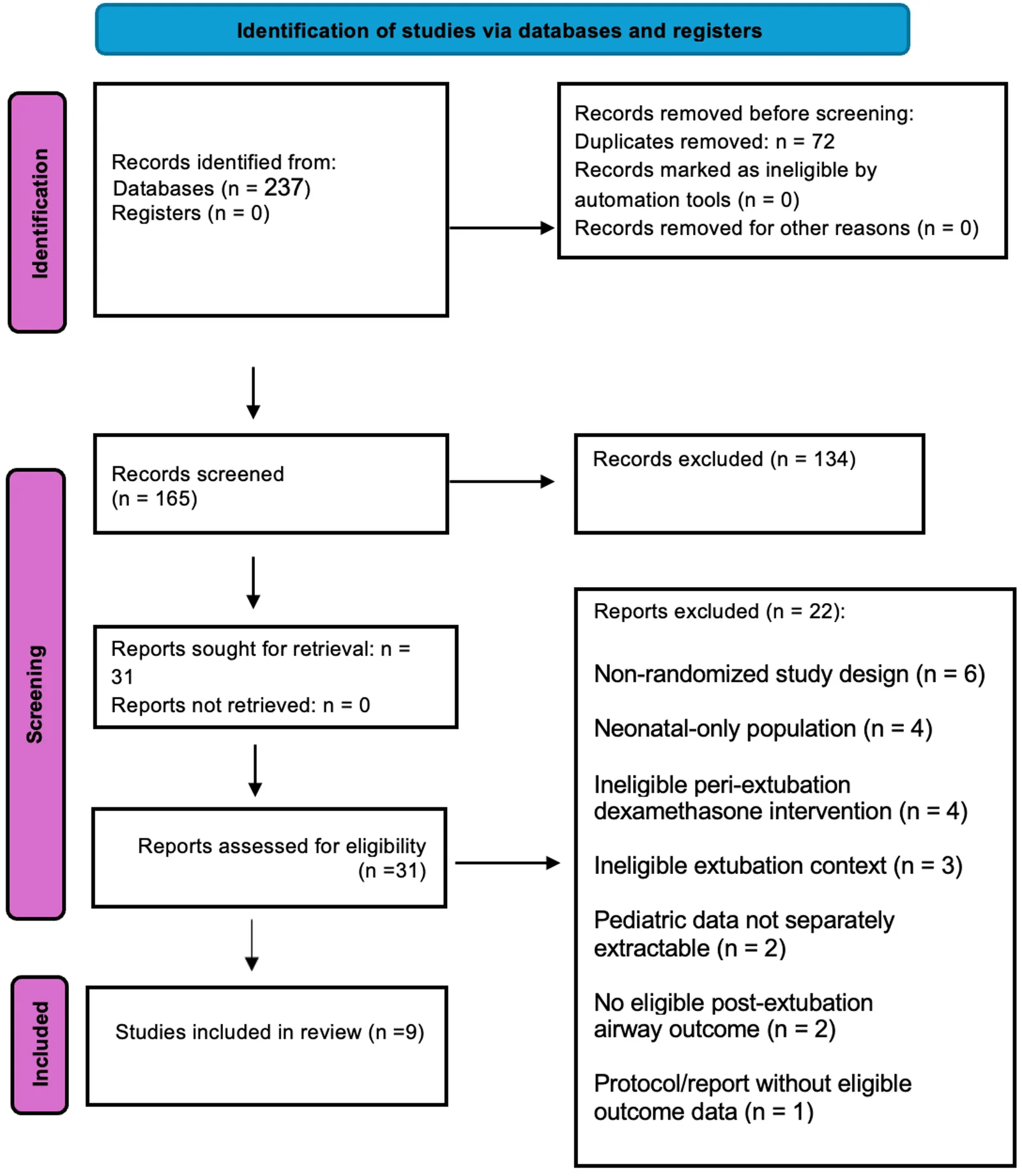
Prisma 2020 Flow Diagram of the Study Selection Process The diagram summarizes study identification, screening, eligibility assessment, and inclusion in the qualitative syntheses, adapted from the PRISMA 2020 statement [9]. PRISMA, Preferred Reporting Items for Systematic Reviews and Meta-Analyses.

Characteristics of Included Trials

Nine randomized trials published between 1991 and 2024 allocated 1,063 children and included 949 participants in the reported analyses. The included trials were conducted in the United States (Tellez et al. [11] and Harel et al. [12]), Brazil (Cesar and de Carvalho [13]), India (Malhotra et al. [14], Baranwal et al. [15], Ritu and Jhamb [16], and Parajuli et al. [17]), Spain (Butragueño-Laiseca et al. [18]), and India (Varghese et al. [19]). Butragueño-Laiseca et al. [18] was conducted across five pediatric intensive care units, whereas Tellez et al. [11] and Harel et al. [12] included two centers each; the remaining trials were single-center studies. Analyzed sample sizes ranged from 23 to 238 children. Eight trials enrolled children exclusively, while Malhotra et al. [14] randomized adults and children separately and reported the pediatric stratum independently. The design and population characteristics of the included trials are summarized in Table 3.

**Table 3:** Characteristics of Included Randomized Trials ICU, intensive care unit; ITT, intention to treat; NR, not reported; PICU, pediatric intensive care unit; SEM, standard error of the mean.

Study	Country, setting, and centers	Design and blinding		Randomized/analyzed	Age	Intubation eligibility and observed duration	Extubation context	Comparator architecture
Tellez et al., 1991 [11]	United States; PICU; two centers		Randomized, double-blind, stratified	153/153; analysis population NR	Mean ± SEM: 2.1 ± 0.4 vs 2.9 ± 0.5 years	No minimum; reported means: 72.8 ± 1.1 vs 84.6 ± 1.1 h after log transformation	Not restricted; repeat intubation during the study period incorporated into the airway-trauma stratum	Dexamethasone vs isotonic saline
Harel et al., 1997 [12]	United States; PICU; two centers		Randomized, double-blind	26/23 in primary analysis; ITT analysis also reported	Mean: 34.6 ± 32 months	No minimum; reported individually for first and second intubations	Subsequent extubation after failure of an initial extubation for post-extubation stridor	Dexamethasone vs sodium chloride
Cesar and de Carvalho, 2009 [13]	Brazil; PICU; single center		Randomized four-group, double-blind	100 allocated/64 matched and analyzed	1 day to 12 years; distribution NR	No minimum; matching used <24 vs ≥24 h	NR	Dexamethasone and/or nebulized L-epinephrine vs corresponding placebos
Malhotra et al., 2009 [14]	India; ICU; single center		Randomized, double-blind; pediatric stratum separately randomized and reported	60 children/60 children; analysis population NR	Mean: 7.87 ± 3.85 vs 7.70 ± 3.93 years	>24 h required; >72 h in 40% vs 50%	First extubation; previous extubation during the same hospitalization excluded	Dexamethasone vs saline
Baranwal et al., 2014 [15]	India; PICU; single center		Randomized, double-blind, stratified	140/124; per protocol	Median: 1.50 (0.41 to 7.0) vs 2.50 (0.75 to 6.0) years	≥48 h required; median 8.0 vs 9.5 days	First planned extubation	24-h vs 6-h dexamethasone regimen
Ritu and Jhamb, 2020 [16]	India; PICU; single center		Randomized, double-blind, stratified	80/80; analysis population NR	Median: 14.5 (2 to 144) vs 11.5 (2 to 132) months	≥48 h required; median ventilation 6 vs 4 days	First extubation; previous failed extubation excluded	Dexamethasone vs normal saline
Parajuli et al., 2021 [17]	India; PICU; single center		Randomized, open-label, stratified, non-inferiority	287/238; per protocol	Median: 33.5 (10 to 72) vs 25 (9 to 72) months	≥48 h required; median 144 h in both arms	First planned extubation	Dexamethasone 0.25 vs 0.5 mg/kg per dose
Butragueño-Laiseca et al., 2022 [18]	Spain; PICU; 5 centers		Randomized, double-blind, multicenter	147/137; per protocol	Median: 7 (2.5 to 54.0) vs 9 (4.0 to 30.0) months	>48 h required; median 8.0 vs 7.0 days	First extubation; previous extubation failure during PICU stay excluded	Dexamethasone vs normal saline
Varghese et al., 2024 [19]	India; PICU; single center		Randomized, double-blind, stratified	70/70; intention to treat	Median: 48 (4.5 to 120) vs 24 (7 to 96) months	>24 h and ≤14 days required; median ventilation 6.5 vs 4 days	NR	Dexamethasone vs normal saline

Seven trials compared dexamethasone with a control intervention; six evaluated planned extubation, whereas Harel et al. [12] evaluated a subsequent extubation attempt after previous extubation failure (Tellez et al. [11], Harel et al. [12], Cesar and de Carvalho [13], Malhotra et al. [14], Ritu and Jhamb [16], Butragueño-Laiseca et al. [18], and Varghese et al. [19]). Two trials directly compared dexamethasone regimens without a placebo arm (Baranwal et al. [15] and Parajuli et al. [17]). Parajuli et al. [17] used an open-label non-inferiority design, whereas the remaining trials reported double blinding.

Analytical approaches differed across trials. Varghese et al. [19] used intention-to-treat analysis. Three trials used per-protocol analyses with post-randomization exclusions (Baranwal et al. [15], Parajuli et al. [17], and Butragueño-Laiseca et al. [18]). These exclusions were related to deferred or altered extubation, non-post-extubation airway obstruction reintubation, protocol violations, or inadequate data collection. The excluded participants represented 16/140, 49/287, and 10/147 randomized participants, respectively. Cesar and de Carvalho [13] analyzed 64 of 100 allocated participants after excluding unmatched participants. Harel et al. [12] excluded three randomized children who received non-randomized dexamethasone but also reported an intention-to-treat analysis. Tellez et al. [11], Malhotra et al. [14], and Ritu and Jhamb et al. [16] did not clearly specify the analysis population.

Four trials did not achieve their planned sample-size targets. Tellez et al. [11] stopped after an interim analysis with 153 of a planned 200 participants. Baranwal et al. [15] randomized 140 participants compared with a calculated requirement of 144. Parajuli et al. [17] enrolled 287 of the planned 526 participants and reported a post hoc power of below 60%. Butragueño-Laiseca et al. [18] randomized 147 participants, compared with an expected 110 per treatment arm, citing recruitment limitations and the expiry of the authorized study period.

Sources of Non-comparability Across Trials

Clinical and methodological comparability was limited by differences in population selection, dexamethasone exposure, endpoint construction, and post-extubation management. Intubation duration eligibility ranged from no minimum requirement in Tellez et al. [11] and Cesar and de Carvalho [13], to >24 hours in Malhotra et al. [14] and Varghese et al. [19], and ≥48 hours in Baranwal et al. [15], Ritu and Jhamb [16], Parajuli et al. [17], and Butragueño-Laiseca et al. [18]. Eligibility restrictions related to neurologic function also varied: Baranwal et al. [15] and Parajuli et al. [17] excluded children with impaired airway reflexes or low Glasgow Coma Scale scores, whereas Varghese et al. [19] excluded pseudobulbar palsy. Harel et al. [12] represented a distinct population, enrolling children undergoing a subsequent extubation attempt after previous extubation failure attributed to post-extubation stridor.

Dexamethasone exposure varied in dose, timing, number of doses, and cumulative pre-extubation exposure (Table 4). Dose per administration ranged from 0.15 to 0.5 mg/kg. Where reconstructable, nominal strictly pre-extubation exposure ranged from 0.2 to 2.5 mg/kg and could not be reliably determined from the published schedules in three trials [11,16,18]. The Baranwal et al. comparison was multidimensional: the 24-hour regimen delivered five dexamethasone doses before extubation compared with one pre-extubation dose in the six-hour regimen, in addition to differences in treatment initiation and cumulative exposure [15]. In Parajuli et al., although five pre-extubation doses were planned, patients received a median of four, and 36/238 (15%) received three or fewer before extubation [17].

**Table 4:** Dexamethasone Regimens and Outcome Ascertainment Across Included Randomized Trials ICU, intensive care unit; NR, not reported; PEAO, post-extubation airway obstruction; UAO, upper airway obstruction.

Study	Dose per administration (maximum)	First dose before extubation	Planned doses	Nominal total planned exposure, mg/kg	Nominal strictly pre-extubation exposure, mg/kg	Endpoint, instrument, and threshold	Outcome assessor and blinding information	Rescue treatment role	Observation window
Tellez et al., 1991 [11]	0.5 mg/kg (10 mg)	6-12 h	6	3.0	Not reconstructable	Significant post-extubation stridor; clinical criteria; non-positional stridor with dyspnea at rest requiring racemic epinephrine	ICU attending/fellow; assessor blinding not separately stated	Racemic epinephrine incorporated into endpoint definition	NR
Harel et al., 1997 [12]	0.5 mg/kg (15 mg)	6 h	4	2.0	0.5	Maximum stridor score; study-specific continuous 11-point score	NR; blinding maintained until study completion	NR	16 h
Cesar and de Carvalho, 2009 [13]	0.2 mg/kg (volume cap 1.3 mL)	1 h	NR	Not reconstructable	0.2	Post-extubation laryngeal edema; Westley Clinical Scoring System; score >0	Double-blind; pharmacist aware of ampoule contents only	Nebulized L-epinephrine randomized as co-intervention	24 h
Malhotra et al., 2009 [14]	0.5 mg/kg (8 mg)	4 h	4	2.0	0.5	Laryngeal edema, dyspnea, and stridor; clinical assessment with separate scoring approach	Assessor blinded	NR	24 h
Baranwal et al., 2014 [15]	0.5 mg/kg (8 mg), both regimens	24 h vs 6 h	6 vs 3	3.0 vs 1.5	2.5 vs 0.5	Clinically significant PEAO; modified Westley score ≥4 requiring nebulized epinephrine and/or reintubation	Trained PICU fellows; agreement not assessed	Nebulized epinephrine incorporated into endpoint; reintubation protocolized	48 h
Ritu and Jhamb, 2020 [16]	0.15 mg/kg	6-12 h or more	6	0.9	Not reconstructable	Post-extubation stridor; any inspiratory sound; Westley score >4 reported separately	Single observer	Westley score >4 triggered nebulized epinephrine; rescue dexamethasone permitted	72 h stated; scoring described to 24 h
Parajuli et al., 2021 [17]	0.25 mg/kg vs 0.5 mg/kg	24 h	6 vs 6	1.5 vs 3.0	1.25 vs 2.5	PEAO; Westley score >4 and/or reintubation for obstruction	At least two clinicians; open-label; investigators not involved in monitoring	Severity-based nebulized epinephrine protocol	24 h
Butragueño-Laiseca et al., 2022 [18]	0.25 mg/kg (8 mg)	6-12 h	4	1.0	Not reconstructable	Stridor and moderate-to-severe UAO; modified Taussig score; stridor or score ≥5	Double-blind; assessor details NR	Rescue treatment based on attending clinician judgment; not standardized	48 h
Varghese et al., 2024 [19]	0.5 mg/kg	12 h	4	2.0	1.0	Clinically significant stridor; Westley stridor score ≥3	Duty doctor, nurses, and investigator blinded; agreement not measured	No routine nebulized epinephrine or budesonide	24 h

Different endpoint definitions yielded different event frequencies within the same randomized cohorts. In Ritu and Jhamb [16], 21/38 (55.2%) placebo-treated children met the broad definition of post-extubation stridor, whereas 18/38 (47.3%) had a Westley score >4. In Butragueño-Laiseca et al. [18], stridor occurred in 28/67 (41.8%) placebo recipients, while 24/67 (35.8%) met the threshold for moderate-to-severe upper airway obstruction. Across trials, airway outcomes were variously defined as post-extubation stridor, laryngeal edema, clinically significant post-extubation airway obstruction, or moderate-to-severe upper airway obstruction and were assessed using clinical criteria, Westley-based scores, a modified Taussig score, or study-specific instruments.

Outcome-assessment windows were also inconsistent. Stated follow-up periods ranged from 16 hours in Harel et al. [12] to 72 hours in Ritu and Jhamb [16], although serial Westley scoring in the latter was described through 24 hours. Tellez et al. [11] did not clearly specify a fixed post-extubation observation window. Post-randomization corticosteroid exposure and rescue treatment practices also differed across trials. Rescue dexamethasone was administered to 6/42 dexamethasone-treated and 13/38 placebo-treated children in Ritu and Jhamb [16]; additional intravenous corticosteroids were given to 7/70 and 11/67 participants, respectively, in Butragueño-Laiseca et al. [18]; and three placebo-assigned children in Harel et al. [12] received non-randomized dexamethasone during follow-up.

Randomized Findings Stratified by Comparator Architecture

Randomized findings according to comparator architecture are summarized in Table 5. In placebo or no-prophylaxis comparisons, dexamethasone did not significantly reduce binary airway obstruction outcomes in Tellez et al. [11], Ritu and Jhamb [16], Butragueño-Laiseca et al. [18], or Varghese et al. [19]. Cesar and de Carvalho [13] reported airway obstruction events in the dexamethasone-alone and double-placebo groups; however, no pairwise effect estimate or statistical comparison was provided. The airway outcome reported by Malhotra et al. [14] was not included in the comparative interpretation because of internally inconsistent reporting.

**Table 5:** Randomized Findings According to Comparator Architecture and Airway Outcomes ARR, absolute risk reduction; CI, confidence interval; ITT, intention-to-treat; NNT, number needed to treat; NR, not reported; OR, odds ratio; PEAO, post-extubation airway obstruction; RR, relative risk; UAO, upper airway obstruction.

Comparison category	Study	Primary airway obstruction endpoint	Main randomized estimate	Secondary severity and rescue outcomes	Reintubation or extubation failure
Placebo/no prophylaxis	Tellez et al., 1991 [11]	Significant post-extubation stridor: 16/76 (21.1%) vs 23/77 (29.9%)	RR: 0.63 (95% CI: 0.30-1.31), p=0.21; adjusted RR: 0.64 (95% CI: 0.30-1.35), p=0.24	Mean racemic-epinephrine treatments: 0.82 vs 0.41, p=0.14	9/76 vs 4/77, p=0.26
Placebo/no prophylaxis	Cesar and de Carvalho, 2009 [13]	Airway obstruction: 10/16 (62.5%) vs 12/16 (75.0%) in dexamethasone-alone vs double-placebo groups	No pairwise estimate or test reported; global four-group χ²=0.58, p>0.05	No four-group difference in maximum score	2/64 overall; arm attribution unclear
Placebo/no prophylaxis	Malhotra et al., 2009 [14]	Internally inconsistent airway endpoint; not used for comparative interpretation	Airway endpoint not interpretable	NR	Failed extubation: 9/30 vs 19/30, p=0.019; interpretation limited
Placebo/no prophylaxis	Ritu and Jhamb, 2020 [16]	Post-extubation stridor: 18/42 (42.8%) vs 21/38 (55.2%)	OR: 0.60 (95% CI: 0.25-1.47), p=0.26; adjusted p=0.17	Median Westley score: 2 vs 2; score >4: 12/42 vs 18/38; adrenaline: 16/42 vs 18/38, p=0.40	7/42 vs 6/38, p=0.90
Placebo/no prophylaxis	Butragueño-Laiseca et al., 2022 [18]	Moderate-to-severe UAO: 16/70 (22.9%) vs 24/67 (35.8%), p=0.095; stridor: 24/69 (34.8%) vs 28/67 (41.8%), p=0.400	No effect estimate reported for primary outcomes	Maximum Taussig score: 2 vs 3, p=0.103; early trajectory difference at 1 h; selected rescue treatment differences	All-cause: 16/70 vs 11/67, p=0.344; UAO-attributed: 3/70 vs 6/67, p=0.319
Placebo/no prophylaxis	Varghese et al., 2024 [19]	Clinically significant stridor: 9/35 (25.7%) vs 11/35 (31.4%), p=0.792	RR: 1.22 (95% CI: 0.580-2.577); absolute risk difference: 5.72 percentage points	Among children with stridor at 6 h, score ≥3: 2/16 vs 7/14, p=0.046	4/35 vs 6/35, p=0.734
Head-to-head regimen	Baranwal et al., 2014 [15]	Clinically significant PEAO: 43/66 (65.2%) vs 48/58 (82.8%), p=0.027	ARR: 17%; NNT: 5.7; corrected RR: 0.79 (95% CI: 0.64-0.97)	Severity trajectory interaction p=0.0001; time to score ≤2, p=0.016; adrenaline requirement among non-reintubated children reduced	5/66 (7.6%) vs 9/58 (15.5%); corrected RR: 0.49 (95% CI: 0.17-1.37)
Head-to-head regimen	Parajuli et al., 2021 [17]	PEAO: 41/121 (33.9%) vs 37/117 (31.6%)	Risk difference: 0.02 (90% CI: −0.07 to 0.12); non-inferiority not demonstrated	No difference in Westley trajectory or recovery time	UAO-attributed reintubation: 10/121 vs 9/117, p=0.87
Repeat extubation evidence	Harel et al., 1997 [12]	Maximum stridor score	No comparative statistical test reported	Maximum score: 5.08 ± 2.84 vs 6.18 ± 3.37, p=0.60; ITT: 5.08 vs 6.07, p=0.64	Extubation failure: 3/12 vs 5/11

Secondary severity outcomes and rescue treatment requirements showed heterogeneous findings. Butragueño-Laiseca et al. [18] reported early differences in the Taussig score trajectory and selected rescue treatment use, whereas maximum score and overall rescue requirements did not differ. Varghese et al. [19] reported lower severity scores among the subgroup of children with stridor at six hours than the entire randomized cohort. Ritu and Jhamb [16] found no significant difference in repeated Westley scores or adrenaline requirement, and Tellez et al. [11] reported no difference in racemic epinephrine treatment frequency.

Reintubation outcomes showed no consistent treatment effect. Tellez et al. [11], Ritu and Jhamb [16], and Varghese et al. [19] reported no significant differences in reintubation. Butragueño-Laiseca et al. [18] reported numerically different patterns for all-cause and airway obstruction-attributed reintubation without statistical significance. Cesar and de Carvalho [13] reported two reintubations overall without reliable arm attribution, whereas failed extubation results from Malhotra et al. [14] were not considered interpretable because of inconsistent airway outcome reporting.

Butragueño-Laiseca et al. [18] reported a prespecified subgroup analysis among children aged ≤2 years, while the analysis of children intubated for >5 days was post hoc. Neither subgroup analysis included a formal treatment-by-subgroup interaction test. Among head-to-head regimen comparisons, Baranwal et al. [15] compared two dexamethasone regimens differing simultaneously in timing, number of pre-extubation doses, and cumulative exposure. The 24-hour regimen was associated with lower clinically significant post-extubation airway obstruction, improved severity trajectory, and lower adrenaline requirement among non-reintubated children. 

Parajuli et al. [17] compared low- versus high-dose dexamethasone using the same planned dosing schedule. The lower-dose regimen did not demonstrate non-inferiority within the prespecified margin, and no significant differences were observed in airway obstruction, reintubation, or recovery-related outcomes. Harel et al. [12] evaluated a distinct repeat-extubation population after previous extubation failure due to post-extubation stridor. Maximum stridor scores did not differ significantly between groups. Extubation failure occurred in 3/12 versus 5/11 children, with no formal between-group statistical test reported. Because of differences in population characteristics, extubation context, and outcome measurement, these findings were considered separately from planned extubation trials.

Airway Obstruction Endpoints, Rescue Therapy, and Reintubation

Across trials reporting multiple levels of the airway obstruction pathway, differences in airway scores or rescue treatment use were not consistently associated with differences in reintubation outcomes. Baranwal et al. [15] reported differences in clinically significant airway obstruction, severity trajectory, and adrenaline requirement between dexamethasone regimens, whereas the reintubation estimate remained imprecise. Butragueño-Laiseca et al. [18] reported early differences in the Taussig-score trajectory and selected rescue treatment use, but neither the primary airway outcome nor the reintubation outcome differed significantly.

Tellez et al. [11] and Ritu and Jhamb [16] demonstrated no corresponding differences across airway outcomes, rescue therapy, and reintubation. Varghese et al. [19] reported a conditional difference in stridor severity among children with stridor at six hours but not in the primary binary airway endpoint or reintubation. In the Parajuli et al. [17] dose comparison, no significant differences were observed in airway obstruction, severity trajectory, recovery time, or reintubation, and non-inferiority of the lower-dose regimen was not demonstrated.

Interpretation of reintubation was limited by variation in outcome attribution and analysis approaches. Butragueño-Laiseca et al. [18] explicitly tabulated both all-cause and upper airway obstruction-attributed reintubation by randomized group, with different numerical directions and no statistically significant difference. Ritu and Jhamb [16] reported all-cause reintubation with aggregate cause attribution, with only one of 13 reintubations attributed to post-extubation stridor. Parajuli et al. [17] excluded non-post-extubation airway obstruction reintubations from the primary per-protocol analysis and subsequently performed a sensitivity analysis including these events. Reintubation criteria were protocolized in several trials, including Baranwal et al. [15], Malhotra et al. [14], Ritu and Jhamb [16], and Parajuli et al. [17], but adjudication of reintubation cause was not reported.

No trial used reintubation as the primary outcome for sample size calculation. Sample size assumptions were instead based on airway obstruction, stridor incidence, or severity measures. Post-randomization exclusions in per-protocol analyses further limited interpretation when clinically unstable children were excluded because of deferred extubation or non-airway reintubation.

Safety and Adverse Event Ascertainment

Safety assessment varied substantially in scope, follow-up duration, and intensity across trials. Baranwal et al. [15] actively monitored gastrointestinal bleeding, hyperglycemia, hypertension, and infection for 48 hours and reported one episode of hyperglycemia requiring short-term insulin therapy. Parajuli et al. [17] followed similar adverse event categories for five days after the final dose and reported one death from massive hematemesis in a child with dengue-related coagulopathy; no hypertension, hyperglycemia, or infection was reported during follow-up.

Ritu and Jhamb [16] used scheduled surveillance for glucose, blood pressure, and gastrointestinal bleeding and reported no gastrointestinal bleeding or hypertension, with similar mean glucose values between groups. Butragueño-Laiseca et al. [18] monitored hypertension, hyperglycemia, gastrointestinal bleeding, and infection; one child required insulin, although baseline glucose values were higher in the dexamethasone group before extubation. Varghese et al. [19] assessed tachycardia, hypertension, hyperglycemia, and gastrointestinal bleeding and reported no significant between-group differences; however, some cardiovascular findings were difficult to attribute specifically to dexamethasone because of concomitant withdrawal treatment.

Tellez et al. [11], Harel et al. [12], Cesar and de Carvalho. [13], and Malhotra et al. [14] did not report dedicated adverse event outcomes. Therefore, the absence of reported adverse events in these trials was interpreted as insufficient safety information rather than as evidence of absence of harm.

Risk-of-Bias Assessment

Risk of bias was assessed using the Cochrane RoB 2 tool and is presented in Figure 2. Overall, seven trials were judged as having some concerns and two as having a high risk of bias; no trial was judged at low overall risk of bias. Cesar and de Carvalho [13] and Malhotra et al. [14] were rated at high overall risk. Across the remaining trials, concerns were most related to the randomization process, deviations from intended interventions, and selection of the reported result, whereas missing outcome data and outcome measurement were generally less problematic.

**Figure 2:**
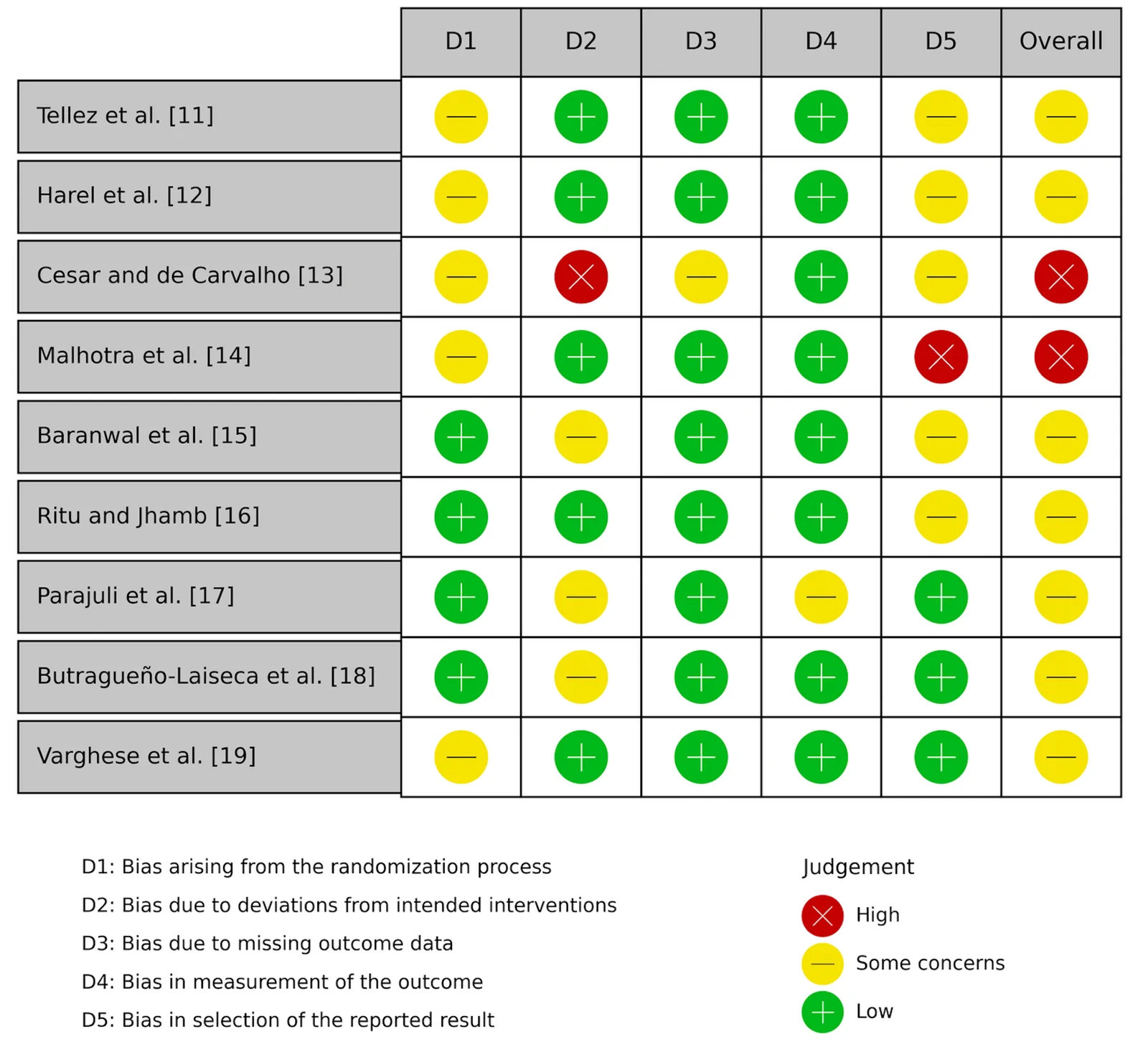
Risk-of-Bias Assessment for the Principal Post-extubation Airway Obstruction Result Across the Included Randomized Trials Using the Cochrane Risk of Bias 2 Tool D1, bias arising from the randomization process; D2, bias due to deviations from intended interventions; D3, bias due to missing outcome data; D4, bias in measurement of the outcome; D5, bias in selection of the reported result. Green indicates low risk of bias, yellow indicates some concerns, and red indicates high risk of bias.

Discussion

Across nine randomized trials spanning more than three decades, the pediatric evidence on peri-extubation dexamethasone does not constitute a single clinically uniform treatment question. Trials differed in eligibility by intubation duration, airway reflexes, and previous extubation failure; in dexamethasone dose, timing, and number of doses delivered before extubation; in comparator architecture; in airway event definitions and thresholds; in rescue treatment practices; and in the analysis populations from which estimates were derived [11-19]. These differences determine the clinical question addressed by each randomized comparison. Given this non-comparability, findings were synthesized according to comparator architecture and sources of non-comparability, with reporting informed by guidance for synthesis without meta-analysis [20].

Among placebo-controlled trials with interpretable binary airway outcomes and pairwise comparisons, no trial provided statistically significant evidence of a reduction in the principal binary airway obstruction outcome [11,16,18,19]. These trials were generally small, several did not achieve their planned sample sizes, and absence of statistical significance should not be interpreted as evidence of no effect [11,15,17,18]. Differences were observed at other levels of the outcome pathway, including severity trajectories, rescue treatment use, and a prespecified age subgroup, although the subgroup finding lacked a formal interaction test and therefore cannot establish treatment effect modification [15,18,19]. The head-to-head trials addressed different questions. The 24-hour versus 6-hour comparison varied initiation time, number of pre-extubation doses, and cumulative exposure simultaneously, whereas the low-dose versus high-dose comparison did not establish non-inferiority of the lower-dose regimen [15,17]. The latter does not demonstrate inferiority of the lower dose or equivalence between regimens. Evidence from repeat extubation after previous failure due to stridor represents a clinically distinct population and was therefore considered separately [12].

The exposure data further limit interpretation of regimen labels. Dose per administration, number of doses delivered before extubation, cumulative pre-extubation exposure, and total planned exposure are distinct treatment dimensions. In the 24-hour versus 6-hour comparison, five doses were scheduled before extubation in one arm compared with one in the other, so any observed difference cannot be attributed to timing alone [15]. Actual pre-extubation dose delivery was incompletely reported across trials; in the low-dose versus high-dose trial, delivered exposure was lower than planned for many participants [17]. For several studies, exposure could therefore be characterized only from nominal schedules rather than observed dose delivery [11,16,18]. The available evidence does not permit reliable identification of an optimal timing, dose, or cumulative regimen.

Outcome construction created a parallel source of non-comparability. Post-extubation stridor, laryngeal edema, clinically significant upper airway obstruction, moderate-to-severe obstruction, rescue treatment requirement, and reintubation represent related but non-equivalent outcomes [11-19]. Different definitions produced different event frequencies even within the same randomized cohorts [16,18]. This distinction extends beyond the included trials; a recent pediatric systematic review treated post-extubation stridor, treatment-requiring upper airway obstruction, laryngoscopic injury, and laryngotracheal stenosis as separate constructs with substantially different reported incidence ranges [21]. Rescue treatment further complicated interpretation because it formed part of the endpoint definition in some trials, was triggered by severity thresholds in others, and was left to clinician judgment elsewhere [11,15-18]. Reintubation is clinically consequential but is not specific to upper airway obstruction. One trial explicitly tabulated both all-cause and upper airway obstruction-attributed reintubation by randomized group, whereas another reported all-cause reintubation with aggregate cause attribution [16,18]. Formal adjudication of reintubation cause was generally not reported, and no included trial was powered primarily for this outcome [11-19].

The available evidence does not support recommending a single peri-extubation dexamethasone regimen, nor does it establish the absence of prophylactic benefit. Future trials should clearly define intubation-duration eligibility, previous extubation failure, impaired airway reflexes, and pre-existing airway pathology. Airway outcomes should distinguish stridor from clinically significant upper airway obstruction, use prespecified severity thresholds, and specify observation windows. Exposure should be reported as the number and timing of doses received before extubation and as the cumulative pre-extubation dose rather than by regimen label alone. Rescue protocols should be prespecified separately from endpoint definitions, and reintubation should have predefined attribution criteria with independent adjudication. Analyses should preserve randomized allocation, with complementary per-protocol analyses where appropriate. Pediatric critical care core outcome initiatives have established common outcome domains for clinical research [22]. An analogous airway-specific outcome framework could improve comparability across peri-extubation trials.

Current pediatric ventilator-liberation guidance conditionally suggests dexamethasone at least six hours before extubation in children considered at high risk of post-extubation upper airway obstruction, but the recommendation is based on very low-certainty evidence [23]. The present findings help define why that certainty remains limited. Timing is difficult to separate from the number of doses and cumulative pre-extubation exposure, and the airway outcomes used across trials are not interchangeable [15-18]. Larger randomized trials using standardized airway endpoints, explicit exposure reporting, and prospectively defined risk groups are required before a specific dose and timing strategy can be established.

Several limitations should be considered. Only nine randomized trials met the eligibility criteria, several were underpowered relative to their planned sample sizes, and none was judged at low overall risk of bias for the principal airway result [11-19]. Clinical and methodological non-comparability was judged to make a common pooled estimate clinically difficult to interpret, and meta-analysis was therefore not performed; statistical heterogeneity and small-study effects were consequently not formally assessed. Adverse event ascertainment was inconsistent and insufficient to characterize harms comprehensively [11-19]. Post-randomization exclusions in several trials also limited interpretation when analyzed populations no longer fully preserved randomized allocation [12,13,15,17,18]. The review was not prospectively registered, and the search was restricted to the databases specified in the Methods. Strengths include restriction to randomized evidence, explicit characterization of dexamethasone exposure, separation by comparator architecture, examination of endpoint construction and post-extubation management, and application of RoB 2 to a defined principal airway result rather than to each study.

Randomized evidence on peri-extubation dexamethasone in critically ill children remains difficult to translate into a single treatment strategy, in part because the trials have addressed related rather than identical clinical questions. Differences in populations, dexamethasone exposure, airway outcome definitions, rescue treatment, and reintubation attribution limit interpretation across studies [11-19]. Standardized eligibility criteria, explicit reporting of pre-extubation exposure, consistent airway endpoints, and prospective attribution of reintubation are needed before an optimal regimen can be established. Until then, the evidence is best interpreted according to regimen and trial architecture rather than as a homogeneous body of evidence.

## Conclusions

Randomized evidence on peri-extubation dexamethasone in critically ill children does not currently define a single optimal prophylactic regimen. The available trials address related but clinically non-equivalent questions, with important variation in patient selection, dexamethasone timing and cumulative pre-extubation exposure, comparator architecture, airway outcome definitions, rescue treatment practices, and reintubation attribution. The lack of a consistent statistically significant effect across placebo-controlled trials does not establish absence of benefit, but the current evidence does not support a universal dose or timing strategy. Future trials should standardize eligibility criteria and airway endpoints, report actual pre-extubation exposure, and prospectively distinguish upper airway obstruction-related reintubation from other causes. Until such evidence is available, the existing evidence should be interpreted according to the specific population, regimen, and outcome framework evaluated, rather than as a homogeneous treatment effect.
